# A controlled T7 transcription-driven symmetric amplification cascade machinery for single-molecule detection of multiple repair glycosylases†

**DOI:** 10.1039/d1sc00189b

**Published:** 2021-03-12

**Authors:** Li-juan Wang, Le Liang, Bing-jie Liu, BingHua Jiang, Chun-yang Zhang

**Affiliations:** College of Chemistry, Chemical Engineering and Materials Science, Collaborative Innovation Center of Functionalized Probes for Chemical Imaging in Universities of Shandong, Key Laboratory of Molecular and Nano Probes, Ministry of Education, Shandong Provincial Key Laboratory of Clean Production of Fine Chemicals, Shandong Normal University Jinan 250014 China cyzhang@sdnu.edu.cn; School of Chemistry and Chemical Engineering, Southeast University Nanjing 211189 China; Academy of Medical Sciences, Zhengzhou University Zhengzhou 450000 China bhjiang@zzu.edu.cn

## Abstract

Genomic oxidation and alkylation are two of the most important forms of cytotoxic damage that may induce mutagenesis, carcinogenicity, and teratogenicity. Human 8-oxoguanine (hOGG1) and alkyladenine DNA glycosylases (hAAG) are responsible for two major forms of oxidative and alkylative damage repair, and their aberrant activities may cause repair deficiencies that are associated with a variety of human diseases, including cancers. Due to their complicated catalytic pathways and hydrolysis mechanisms, simultaneous and accurate detection of multiple repair glycosylases has remained a great challenge. Herein, by taking advantage of unique features of T7-based transcription and the intrinsic superiorities of single-molecule imaging techniques, we demonstrate for the first time the development of a controlled T7 transcription-driven symmetric amplification cascade machinery for single-molecule detection of hOGG1 and hAAG. The presence of hOGG1 and hAAG can remove damaged 8-oxoG and deoxyinosine, respectively, from the dumbbell substrate, resulting in breaking of the dumbbell substrate, unfolding of two loops, and exposure of two T7 promoters simultaneously. The T7 promoters can activate symmetric transcription amplifications with the unfolded loops as the templates, inducing efficient transcription to produce two different single-stranded RNA transcripts (*i.e.*, reporter probes 1 and 2). Reporter probes 1 and 2 hybridize with signal probes 1 and 2, respectively, to initiate duplex-specific nuclease-directed cyclic digestion of the signal probes, liberating large amounts of Cy3 and Cy5 fluorescent molecules. The released Cy3 and Cy5 molecules can be simply measured by total internal reflection fluorescence-based single-molecule detection, with the Cy3 signal indicating the presence of hOGG1 and the Cy5 signal indicating the presence of hAAG. This method exhibits good specificity and high sensitivity with a detection limit of 3.52 × 10^−8^ U μL^−1^ for hOGG1 and 3.55 × 10^−7^ U μL^−1^ for hAAG, and it can even quantify repair glycosylases at the single-cell level. Moreover, it can be applied for the measurement of kinetic parameters, the screening of potential inhibitors, and the detection of repair glycosylases in human serum, providing a new paradigm for repair enzyme-related biomedical research, drug discovery, and clinical diagnosis.

## Abstract 2

A controlled T7 transcription-driven symmetric amplification cascade machinery is developed for single-molecule detection of multiple repair glycosylases.

## Graphical abstract

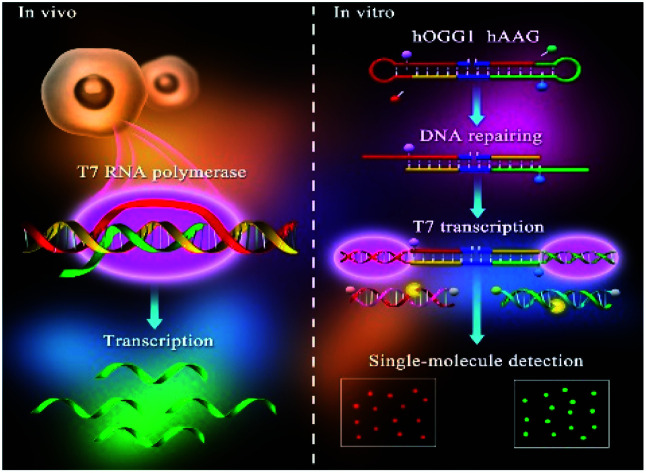

## Introduction

The specific pairing of heterocyclic bases (*i.e.*, A with T, and G with C) in the DNA double helix is critically important for the preservation and transmission of genetic information encoded in human genomes.^1^ However, the chemical structures of heterocyclic bases possess a large number of nucleophilic and redox-active sites, which are frequently attacked by various exogenous (*e.g.*, ultraviolet radiation, genotoxic chemicals, and tobacco smoking)^2,3^ and endogenous insults (*e.g.*, reactive oxygen species and *S*-adenosylmethionine),^4^ inducing a variety of types of oxidative damage (*e.g.*, oxidized bases, abasic sites, and strand breaks)^5^ and alkylative lesions (*e.g.*, alkylated bases, deaminated purines, and cyclic adducts).^6^ Among these, 8-oxo-7,8-dihydro-2′-deoxyguanosine (8-oxodG) is the most abundant oxidative lesion and it can mispair with 2′-deoxyadenosine (dA) during DNA replication to cause permanent G:C → T:A transversion mutations;^7^ the *N*^3^-methyl-2′-deoxyadenosine (m3dA) is the predominant alkylative lesion and it can undergo spontaneous depurination to block most polymerases, thus impeding DNA replication and transcription.^8,9^ Human 8-oxoguanine DNA glycosylase (hOGG1)^10^ and human alkyladenine DNA glycosylase (hAAG)^11^ are two types of repair glycosylases with distinct functions and substrate specificities, and they can catalyze the repair of two types of major oxidative and alkylative damage through the classic base excision repair (BER) mechanism.^12^ The dysregulation of hOGG1 and hAAG is associated with the initiation and progression of various human diseases (*e.g.*, neurodegeneration, immunodeficiency, chronic inflammation, hypoalbuminemia, lymphomas, glioblastoma, leukaemias, and xeroderma pigmentosum),^13,14^ and cancers (*e.g.*, lung, colon, breast, liver, cervix, stomach, gallbladder, bladder, and oropharynx cancers).^15,16^ Therefore, hOGG1 and hAAG may function as both important biomarkers and therapeutic targets, and the simultaneous detection of hOGG1 and hAAG activities is of great significance to DNA damage-related biomedical research and clinical therapeutics.

So far, great efforts have been devoted to developing efficient and sensitive methods for repair glycosylase assays. Traditional methods include gel electrophoresis-coupled radioisotope labeling,^17,18^ enzyme-linked immunoassay (ELISA),^19^ mass spectrometry (MS),^20^ and high-performance liquid chromatography (HPLC).^21^ However, the gel-based radiometric assays suffer from hazardous radiation and time-consuming operations;^17,18^ ELISA requires expensive antibodies and may suffer from underestimation caused by sample losses during multiple washing steps.^19^ Moreover, all these methods are heterogeneous and semi-quantitative.^17–19^ The MS and HPLC methods have high backgrounds resulting from artificial DNA damage during complex sample preparation.^20,21^ Alternatively, several new methods, including colorimetric,^22^ electrochemical,^23^ fluorescent,^10,15,24,25^ and luminescent assays,^26–28^ have been developed. The colorimetric assay enables the visualized detection of hOGG1 activity,^22^ but the preparation of DNA-AuNP probes is time-consuming and laborious. The electrochemical method takes advantage of uracil hydrolysis-initiated DNA duplex unwinding and subsequent guanine oxidation in the released single-stranded DNA (ssDNA) at graphene-deposited electrodes to quantify uracil DNA glycosylase (UDG) activity,^23^ but the preparation of modified electrodes and the immobilization of DNA probes are relatively cumbersome and complicated. The fluorescent assay utilizes 8-oxoG repair-induced assembly of a quantum dot-based nanosensor to detect hOGG1 activity,^10^ but the intricate probe modifications and costly fluorescent nanomaterials limit its wide application. The luminescent assays based on the combination of DNA repair-response cleavage with the G-quadruplex-selective iridium(iii) complex^26,28^ and *in vitro* green fluorescent protein expression^27^ enable the detection of DNA repair enzyme activity^26^ and inhibitor screening,^27,28^ with distinct advantages of easy probe preparation, simple strategy, and low cost compared with the fluorescent assays. However, due to the lack of target amplification, the improvement in sensitivity is not significant for luminescent assays. To improve the sensitivity, some deoxyribonucleotide amplification techniques have been introduced for the detection of repair glycosylase activities, including loop-mediated isothermal amplification (LAMP),^15^ rolling circle amplification (RCA),^24^ and endonuclease (*e.g.*, Fok I)-assisted signal amplification (EASA).^25^ However, these methods involve complicated amplification procedures, multiple primers/enzymes, and high backgrounds resulting from the nonselective fluorescent dyes, nonspecific polymerization and digestion. In addition, all the above methods can only detect one type of repair glycosylase. Taking into account the fact that one disease is usually associated with multiple repair enzymes,^11,15,24,29^ the development of facile, accurate, and sensitive methods for simultaneous detection of multiple repair glycosylases is urgently needed.

Recently, a variety of DNA polymerization amplification techniques, such as polymerase chain reaction (PCR),^30,31^ strand displacement amplification (SDA),^32^ and exponential isothermal amplification reaction (EXPAR),^33,34^ have been introduced into biosensing systems to improve their performance. However, the steric hindrance, variant chemical microenvironment, and surface crowding effect on the interfaces of biosensors may inevitably induce low binding efficiency and enzyme kinetics due to primer-/template-dependent DNA syntheses.^35–37^ Although improvement of interfacial engineering with nanostructures can maximize the target recognition efficiency, the variability in surface micro-/nano-fabrication may significantly affect the quantification and reproducibility in the complicated matrix.^38,39^ Alternatively, transcription amplification is a new RNA polymerization amplification technique based on the repeated polymerization of ribonucleoside triphosphates by T7 RNA polymerase in a 5′ to 3′ direction on double-stranded DNA (dsDNA) only in the presence of a specific T7 promoter region. It enables the transcription of T7-promoter downstream DNA sequence into large amounts of ssRNA sequences under isothermal conditions within 1 h.^40,41^ In comparison with DNA amplification techniques, T7-based transcription amplification has distinct advantages of flexible sequence design, easy interfacial nano-fabrication preparation, high hybridization efficiency, rapid enzyme kinetics, efficient elimination of nonspecific amplification, and no involvement of thermal cycler, and it has become a simple and robust platform for isothermal nucleic acid amplification.^42^ Recently, single-molecule detection has attracted much attention due to its significant advantages of high signal-to-noise ratio, high sensitivity, and low sample consumption.^43–45^ In this research, we demonstrate the development of a controlled T7 transcription-driven symmetric amplification cascade machinery for single-molecule detection of multiple repair glycosylases by taking advantage of the intrinsic superiorities of T7-based transcription amplification and single-molecule detection. This method exhibits good specificity, high sensitivity, with a detection limit of 3.52 × 10^−8^ U μL^−1^ for hOGG1 and 3.55 × 10^−7^ U μL^−1^ for hAAG, and a large dynamic range with five orders of magnitude. It can be applied to measure kinetic parameters, screen potential inhibitors, and quantify repair glycosylases from human serum and even one single lung cancer cell, holding great potential in biomedical research, drug discovery, and clinical diagnosis.

## Results and discussion

**Fig. 1:**
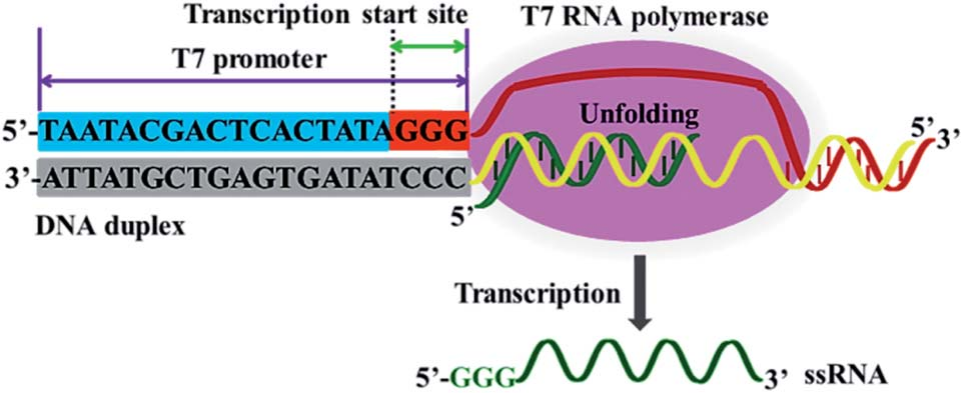
Mechanism of T7-based transcription amplification. T7 RNA polymerase can recognize the specific T7 promoter and catalyze the transcription of downstream DNA sequence into ssRNA sequences.

T7 RNA polymerase from bacteriophage T7 is a DNA-dependent RNA polymerase and it is highly specific for T7 phage promoter.^41^ T7 RNA polymerase shares numerous functional characteristics with cellular RNA polymerases, and it has initiation and elongation phases during the transcription process.^40^ As shown in Fig. 1, at the initiation phase, T7 RNA polymerase can bind to the specific T7 promoter, unfolding the DNA duplex at the transcription start site (*i.e.*, the 5′-GGG-3′ region) (Fig. 1, orange color), and simultaneously activating RNA synthesis *de novo*. At the elongation phase, T7 RNA polymerase can catalyze the repeated polymerization of ribonucleoside triphosphates in a 5′ to 3′ direction to complete the transcription progressively, without dissociation, until termination, efficiently transcribing the downstream DNA sequence into large amounts of ssRNA sequences under the T7 promoter.^41^

**Scheme 1:**
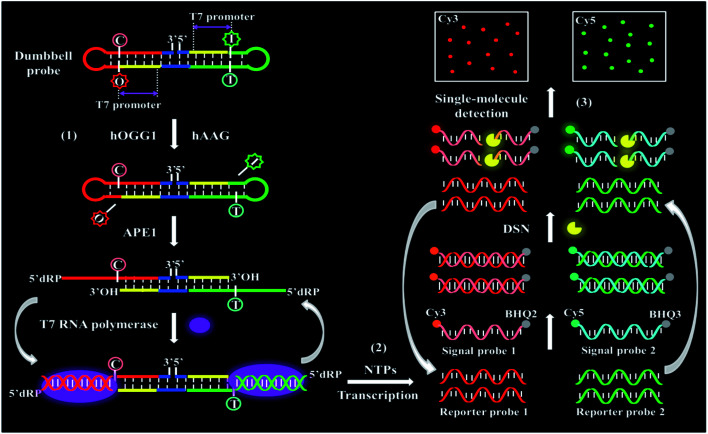
Schematic illustration of a controlled T7 transcription-driven symmetric amplification cascade machinery for single-molecule detection of multiple repair glycosylases.

We demonstrate the development of a controlled T7 transcription-driven symmetric amplification cascade machinery for single-molecule detection of multiple repair glycosylases with hOGG1 and hAAG as the target models. The principle of the proposed repair glycosylases assay is illustrated in Scheme 1. In this strategy, we designed one dumbbell probe and two linear signal probes. The bifunctional dumbbell probe is composed of two domains (*i.e.*, a stem domain and two loop domains), and it functions as both the catalytic substrate for repair glycosylases and the transcription template for T7-based transcription amplifications. The stem contains two complementary strands, including the upper strand and the lower strand. In the upper strand, a damaged deoxyinosine (*i.e.*, I) base (Scheme 1, green color) is designed to be three bases away from the loop 1 structure close to the 5′ end (Scheme 1, green color), and the 20-nucleotide (nt) sequence (Scheme 1, yellow color) at the 5′ end next to the deoxyinosine base is a T7 promoter region. Similarly, in the lower strand, a damaged 8-oxoG (*i.e.*, O) base (Scheme 1, red color) is located three bases away from the loop 2 structure close to the 3′ end (Scheme 1, red color), and the 20-nucleotide sequence (Scheme 1, yellow color) at the 5′ end next to the 8-oxoG base is a T7 promoter region. The damaged 8-oxoG and deoxyinosine bases can be excised by hOGG1 and hAAG, respectively, for simultaneously actuating the repairing-activated T7 transcription-dependent amplification cascades. Both loops 1 and 2 are 9-nt sequences, and they can act as transcription templates to produce different reporter probes 1 and 2 upon unfolding. Both signal probes 1 and 2 (Scheme 1, pink and blue colors) are 10-nt DNA sequences modified by fluorophores (Cy3 or Cy5) at the 5′ end and quenchers (BHQ2 or BHQ3) at the 3′ end, respectively, which can hybridize with reporter probes 1 and 2, respectively, to initiate the duplex-specific nuclease (DSN)-directed cyclic liberation of Cy3 and Cy5 fluorophores. As shown in Scheme 1, this strategy consists of three consecutive steps: (1) the excision of damaged bases catalyzed by repair glycosylases causes the unfolding of two loops in the dumbbell probe; (2) the subsequent repairing-activated T7 transcription-dependent cascade amplification induces the liberation of Cy3 and Cy5 fluorophores; (3) the released Cy3 and Cy5 fluorophores are counted by total internal reflection fluorescence (TIRF)-based single-molecule detection. In the presence of hOGG1 and hAAG, the damaged bases 8-oxoG (Scheme 1, red color) and deoxyinosine (Scheme 1, green color) in the dumbbell probe are specifically recognized and efficiently excised from the 8-oxo G:C and I:T pairs, respectively, through cleaving the C1–N glycosidic bonds connected to the damaged bases, leaving two apurinic (AP) sites.^46^ These two AP sites can be subsequently cleaved by human AP endonuclease (APE1) through the hydrolysis of 5′-phosphodiester bonds, leaving 5′-deoxyribose phosphate (5′-dRP) and 3′-OH termini,^47^ and simultaneously leading to breaking of the dumbbell probe, unfolding of loops 1 and 2, and exposure of two T7 promoter regions. With the unfolded sequences of loops 1 and 2 as the templates, the corresponding T7 promoters in the opposite complementary strands can activate the transcription amplification reactions in the presence of T7 RNA polymerase, inducing efficient transcription of templates to produce large amounts of single-stranded RNA transcripts (*i.e.*, reporter probes 1 and 2), respectively. The resultant reporter probes 1 and 2 can hybridize with the signal probes 1 and 2, respectively, to form two RNA/DNA heteroduplexes 1 and 2, which may function as the favorite substrates of DSN (it can specifically degrade the DNA strand in RNA/DNA hybrid duplexes, but has little activity towards the RNA strand).^25^ Subsequently, the RNA/DNA heteroduplexes 1 and 2 can be progressively digested by DSN, resulting in the releases of Cy3 and Cy5 fluorophores as well as reporter probes 1 and 2. Notably, the released reporter probes 1 and 2 can further hybridize with free signal probes 1 and 2, respectively, to initiate multiple cycles of digestion-release-hybridization, eventually liberating large amounts of Cy3 and Cy5 fluorophores. Through simply monitoring the Cy3 and Cy5 signals by TIRF-based single-molecule imaging, the hOGG1 and hAAG activities can be quantitatively detected. In contrast, in the absence of hOGG1 and hAAG, neither 8-oxoG base nor deoxyinosine base can be removed, and no dumbbell probe can be cleaved, and thus no T7 transcription-dependent cycling cascade amplification can be initiated. As a result, no signal probes can be digested, and neither Cy3 nor Cy5 fluorescence signal can be observed. Taking advantage of the high precision of the natural BER mechanism, the high specificity and efficiency of T7 transcription-dependent cycling cascade amplification, and the high signal-to-noise ratio of single-molecule detection, the proposed strategy provides a facile and robust platform for the simultaneous detection of hOGG1 and hAAG activities with good specificity and high sensitivity.

**Fig. 2:**
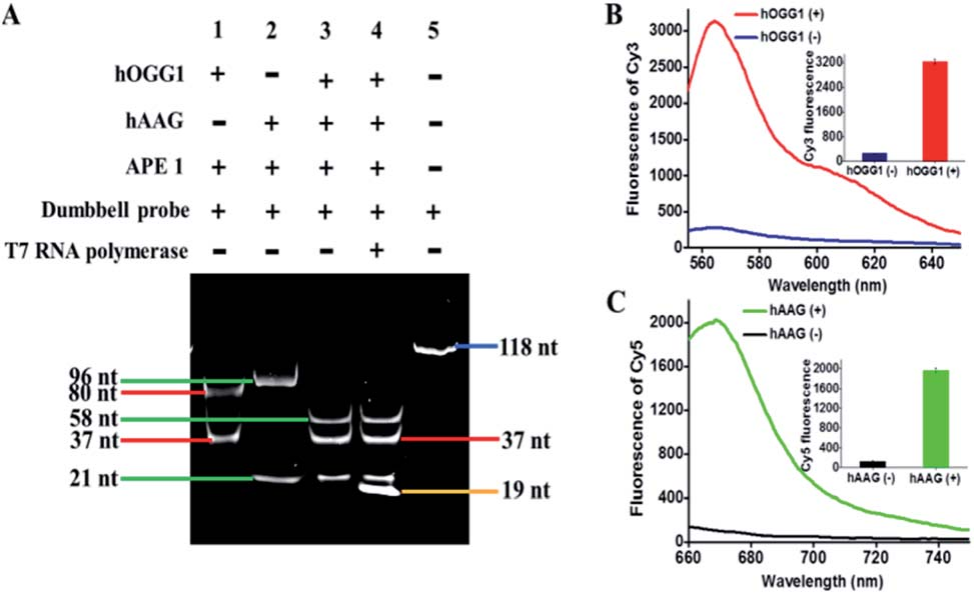
(A) Denaturing PAGE analysis of the products of DNA glycosylase-catalyzed damaged base excision repair and the products of transcription amplification reactions under different conditions. Lane 1, hOGG1 + APE1 + dumbbell probe; lane 2, hAAG + APE1 + dumbbell probe; lane 3, hOGG1 + hAAG + APE1 + dumbbell probe; lane 4, hOGG1 + hAAG + APE1 + dumbbell probe + T7 RNA polymerase; lane 5, the synthetic dumbbell probe. In (A) 0.4 U μL^−1^ hOGG1, 0.4 U μL^−1^ hAAG and 1.5 U μL^−1^ APE1 were used in the experiments. (B) Fluorescence measurements of hOGG1-catalyzed 8-oxoG repairing-activated T7 transcription-dependent amplification cascade-induced release of Cy3 fluorophores in the absence (blue curve) and presence (red curve) of hOGG1. Inset shows the fluorescence intensity of Cy3 in the absence (blue column) and presence (red column) of hOGG1. (C) Fluorescence measurements of hAAG-catalyzed deoxyinosine repairing-activated T7 transcription-dependent amplification cascade-induced release of Cy5 fluorophores in the absence (black curve) and presence (green curve) of hAAG. Inset shows the fluorescence intensity of Cy5 in the absence (black column) and presence (green column) of hAAG. In (B and C), 0.1 U μL^−1^ hOGG1, 0.1 U μL^−1^ hAAG and 0.5 U μL^−1^ APE1 were used in the experiments.

To investigate whether hOGG1 and hAAG can excise 8-oxoG and deoxyinosine to induce the unfolding of loops 1 and 2 in the dumbbell probe, respectively, we utilized 14% denaturing PAGE to analyze the excision products, with SYBR Gold as the fluorescent indicator. As shown in Fig. 2A, two characteristic bands of 80 nt and 37 nt are observed in the presence of hOGG1 + APE1 + dumbbell probe (Fig. 2A, lane 1), which are exactly the sizes of the longer excision product (80 nt) and the shorter excision product (37 nt), indicating that hOGG1 can accurately and efficiently excise the 8-oxoG repair with the assistance of APE1 to generate a nucleotide gap in the dumbbell probe, inducing the cleavage of dumbbell probes into two fragments. Similarly, two characteristic bands (96 nt and 21 nt) are observed in the presence of hAAG + APE1 + dumbbell probe (Fig. 2A, lane 2), which are exactly the sizes of the longer excision product (96 nt) and the shorter excision product (21 nt), indicating that hAAG can specifically excise the deoxyinosine repair with the assistance of APE1 to produce a nucleotide gap for the cleavage of dumbbell probe into two fragments. In the presence of hOGG1 + hAAG + APE1 + dumbbell probe, the excision products with different lengths (*i.e.*, 37 nt, 21 nt, and 58 nt) are observed (Fig. 2A, lane 3), demonstrating that the presence of hOGG1 and hAAG enables the damaged 8-oxoG and deoxyinosine excision repair, respectively, leading to the cleavage of dumbbell probes for the generation of different DNA fragments. Notably, the 58-nt band is the size of the excision product resulting from the simultaneous cleavage of the same dumbbell probe by hOGG1 and hAAG. We further analyzed the reaction products of transcription amplification with the addition of T7 RNA polymerase into the reaction system containing hOGG1 + hAAG + APE1 + dumbbell probes. A distinct band of 19 nt is observed (Fig. 2A, lane 4), which is identical to the sizes of two RNA transcripts (*i.e.*, reporter probes 1 and 2), indicating that the presence of hOGG1 and hAAG can induce the removal of 8-oxoG and deoxyinosine, respectively, and lead to breaking of the dumbbell probe, unfolding of two loops, and exposure of two T7 promoters simultaneously. Subsequently, the T7 promoters activate the transcription amplifications *via* T7 RNA polymerase catalysis with the unfolded loops 1 and 2 as the templates, for the generation of large amounts of 19-nt reporter probes 1 and 2. To further verify the feasibility of the proposed strategy, we added signal probes 1 and 2 into the reaction system containing hOGG1 + hAAG + APE1 + dumbbell probe + T7 RNA polymerase and measured the fluorescence emission spectra under different conditions (Fig. 2B and C). A significant Cy3 fluorescence signal with a characteristic emission peak of 562 nm is detected in the presence of hOGG1 (Fig. 2B, red curve), and a significant Cy5 fluorescence signal with a characteristic emission peak of 665 nm is detected in the presence of hAAG (Fig. 2C, green curve), indicating that hOGG1 and hAAG can catalyze the 8-oxoG and deoxyinosine excision repair, respectively, activating T7 transcription-dependent amplification reactions to produce abundant reporter probes 1 and 2. The resulting reporter probes 1 and 2 can hybridize with signal probes 1 and 2 to induce DSN-directed cyclic liberation of Cy3 and Cy5 fluorescent molecules, respectively. In contrast, no significant Cy3 (Fig. 2B, blue curve) and Cy5 fluorescence signals (Fig. 2C, black curve) are detected in the absence of hOGG1 and hAAG. Notably, no nonspecific bands are detected in the presence of hOGG1 + hAAG + T7 RNA polymerase (Fig. 2A, lane 4), and very low background signals are observed in the absence of hOGG1 (Fig. 2B, blue curve) and hAAG (Fig. 2C, black curve), respectively. This can be ascribed to the following factors: (1) the repair glycosylase-initiated natural BER mechanism exhibits high accuracy towards the damaged bases, inhibiting nonselective excision; (2) the T7-based transcription enables extremely specific amplification of RNA transcript, preventing nonspecific amplification; (3) the DSN possesses high selectivity towards the DNA in RNA/DNA heteroduplexes, inducing the specific digestion of signal probes. These results (Fig. 2) clearly demonstrate that the proposed method can be used for the simultaneous detection of hOGG1 and hAAG.

**Fig. 3:**
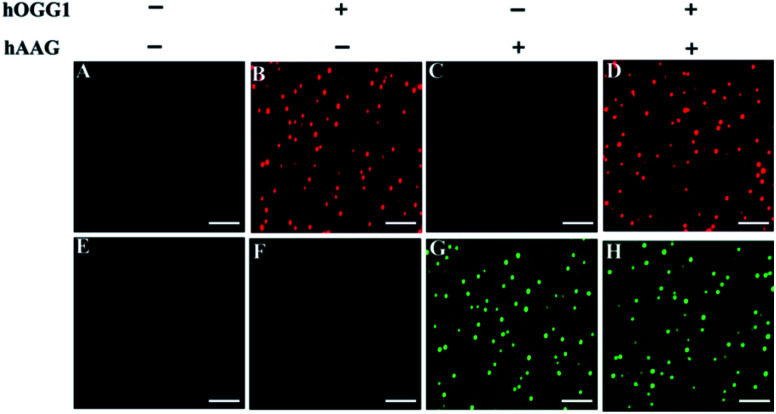
Single-molecule imaging in the absence (A and E) and presence of hOGG1 (B and F), hAAG (C and G), and hOGG1 + hAAG (D and H), respectively. The Cy3 fluorescence signal is shown in red, and the Cy5 fluorescence signal is shown in green. The hOGG1 concentration is 0.1 U μL^−1^, and the hAAG concentration is 0.1 U μL^−1^. The scale bar is 5 μm.

We employed TIRF-based fluorescence imaging to detect repair glycosylase activities at the single-molecule level. TIRF microscopy is based on the total internal reflection phenomenon that occurs when light passes from a high-refractive medium (*e.g.*, glass) into a low-refractive medium (*e.g.*, water) (Reck-Peterson *et al.* 2010).^48^ The evanescent field produced by the total internally reflected light only excites the fluorescent molecules in a thin layer (<100 nm) next to the reflection interface, efficiently minimizing background noise emanating from the inner depths.^49^ As shown in Fig. 3, distinct Cy3 fluorescence signals are detected in the presence of hOGG1 at an excitation wavelength of 561 nm (Fig. 3B), and distinct Cy5 fluorescence signals are observed in the presence of hAAG at an excitation wavelength of 640 nm (Fig. 3G), indicating that hOGG1 and hAAG can catalyze the damaged 8-oxoG and deoxyinosine bases repair, respectively, and initiate the subsequent T7 transcription-dependent amplification cascades to release Cy3 and Cy5 fluorescent molecules, respectively. In contrast, neither Cy3 nor Cy5 fluorescence signals are observed in the absence of hOGG1 (Fig. 3A) or hAAG (Fig. 3E), indicating no occurrence of the repairing-activated T7 transcription-dependent cascade amplifications, and consequently no release of Cy3 and Cy5 fluorescent molecules. The presence of both hOGG1 and hAAG can simultaneously generate both Cy3 (Fig. 3D) and Cy5 (Fig. 3H) fluorescence signals at dual excitation wavelengths of 561 nm and 640 nm. These results clearly demonstrate that the Cy3/Cy5 fluorescence pair is suitable for the simultaneous repair glycosylase assay at the single-molecule level, and the proposed method is capable of detecting the activities of multiple repair glycosylases with high specificity.

**Fig. 4:**
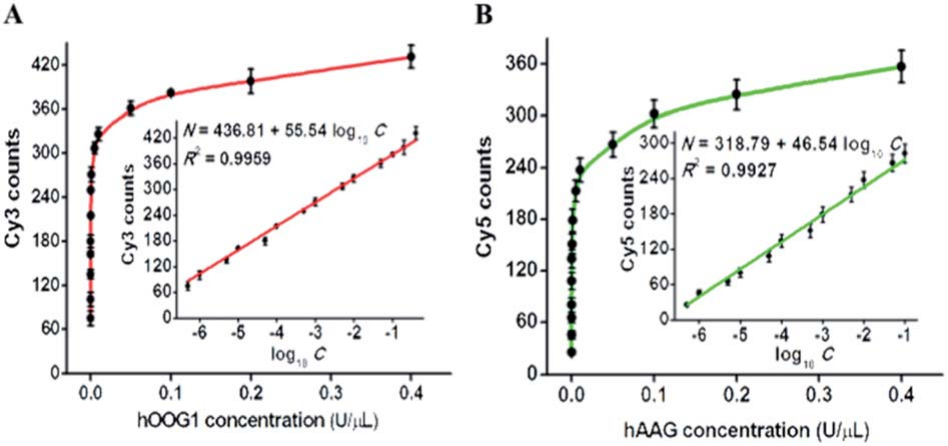
(A) Variance of the Cy3 counts with different concentrations of hOGG1. The inset shows the linear correlation between the Cy3 counts and the logarithm of hOGG1 concentration from 5 × 10^−7^ to 0.4 U μL^−1^. (B) Variance of the Cy5 counts with different concentrations of hAAG. The inset shows the linear correlation between the Cy5 counts and the logarithm of hAAG concentration from 5 × 10^−7^ to 0.1 U μL^−1^. Error bars represent the standard deviations of three independent experiments.

To investigate the detection sensitivity of the proposed method, we optimized the experimental conditions, including the amounts of T7 RNA polymerase, nuclease DSN, and the types and concentrations of signal probes (Fig. S1–S4†). Under optimal reaction conditions, we evaluated the sensitivity of the proposed method by measuring the counts of Cy3 and Cy5 fluorescent molecules in response to different concentrations of hOGG1 and hAAG (Fig. 4). As shown in Fig. 4A, with the increase of hOGG1 concentration from 5 × 10^−7^ to 0.4 U μL^−1^, the Cy3 counts are enhanced in a dose-dependent manner, and a good linear correlation is obtained between the Cy3 counts and the logarithm of hOGG1 concentration over a large dynamic range of five orders of magnitude, from 5 × 10^−7^ to 0.4 U μL^−1^ (inset of Fig. 4A). The regression equation is *N* = 436.81 + 55.54 log_10_ *C* with a correlation coefficient of 0.9959, where *N* is the measured Cy3 count, and *C* is the hOGG1 concentration (U μL^−1^). The detection limit is calculated to be 3.52 × 10^−8^ U μL^−1^ by evaluating three times the standard deviation plus the average response of the negative control. This sensitivity is enhanced by as much as four orders of magnitude compared with that of a terminal-protected DNA-AuNP probe-based colorimetric assay (7.0 × 10^−4^ U μL^−1^),^22^ 62.5-fold compared with that of a DNA repair-responsive molecular beacon-based fluorescent assay (2.2 × 10^−6^ U μL^−1^),^29^ 12.2-fold compared with that of a Fok I-assisted signal amplification-based fluorescent assay (4.3 × 10^−7^ U μL^−1^),^25^ and is comparable with the LAMP-based fluorescent assay (1.0 × 10^−8^ U μL^−1^).^15^ As shown in Fig. 4B, with the increase of hAAG concentration from 5 × 10^−7^ to 0.4 U μL^−1^, the Cy5 counts enhance in a concentration-dependent manner, and a good linear correlation is obtained between the Cy5 counts and the logarithm of hAAG concentration over a large dynamic range of five orders of magnitude, from 5 × 10^−7^ to 0.1 U μL^−1^ (inset of Fig. 4B). The regression equation is *N* = 318.79 + 46.54 log_10_ *C* with a correlation coefficient of 0.9927, where *N* is the measured Cy5 counts, and *C* is the concentration of hAAG (U μL^−1^), respectively. The detection limit is calculated to be 3.55 × 10^−7^ U μL^−1^ by evaluating three times the standard deviation plus the average response of the negative control. This sensitivity is improved by as much as 281.7-fold compared with that of the bead DNA nanoprobe-based fluorescent assay (1 × 10^−4^ U μL^−1^),^50^ 253.5-fold compared with that of the target-directed hyperbranched amplification-based fluorescent assay (9.0 × 10^−5^ U μL^−1^),^51^ and 13.8-fold compared with that of the autocatalytic cleavage-induced fluorescence recovery-based assay (4.9 × 10^−6^ U μL^−1^).^11^ The improved sensitivity of the proposed method can be attributed to: (1) the high accuracy and selectivity of DNA glycosylase-catalyzed damaged base excision repair, (2) the high specificity and efficiency of T7 transcription-dependent cycling cascade amplification, and (3) the high signal-to-noise ratio of single-molecule detection.

**Fig. 5:**
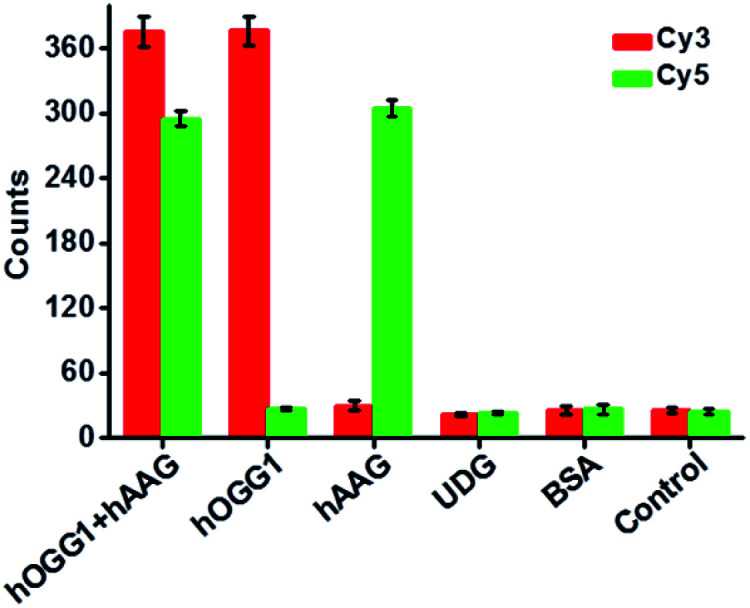
Measurement of Cy3 (red column) and Cy5 (green column) counts in response to 0.1 U μL^−1^ hOGG1 + 0.1 U μL^−1^ hAAG, 0.1 U μL^−1^ hOGG1, 0.1 U μL^−1^ hAAG, 0.1 U μL^−1^ UDG, 0.1 g L^−1^ BSA, and the control without any enzymes, respectively. Error bars represent the standard deviations of three independent experiments.

Repair glycosylase is an enzyme superfamily comprising a large group of members,^15^ and differentiation of one repair glycosylase from other family members has remained a great challenge. To evaluate the selectivity of the proposed method, we used bovine serum albumin (BSA)^10^ and uracil DNA glycosylase (UDG)^33^ as the negative controls. BSA is an irrelevant protein, and it cannot recognize and catalyze the damaged base repair in the dumbbell probe.^10^ UDG is a member of the repair glycosylase family, but it can only locate and excise uracil from the U:A mismatch.^33^ In theory, neither BSA nor UDG can catalyze the damaged base (*i.e.*, 8-oxoG or deoxyinosine) repair to initiate T7 transcription-dependent amplification cascades for the release of Cy3 and Cy5 fluorophores. As shown in Fig. 5, neither a Cy3 nor a Cy5 fluorescence signal is detected in the presence of BSA and UDG, respectively, similar to that of the control group without any enzyme. In contrast, a significant Cy3 fluorescence signal is observed in the presence of hOGG1, and a significant Cy5 fluorescence signal is observed in the presence of hAAG, and both distinct Cy3 and Cy5 fluorescence signals are simultaneously detected in the presence of both hOGG1 and hAAG, indicating that only hOGG1 and hAAG can specifically recognize and catalyze the damaged 8-oxoG and deoxyinosine base repair, respectively, to initiate subsequent T7 transcription-dependent cycling cascade amplification for the release of Cy3 and Cy5 fluorophores. These results clearly demonstrate that the proposed method can discriminate hOGG1 and hAAG from other interference enzymes with high selectivity.

**Fig. 6:**
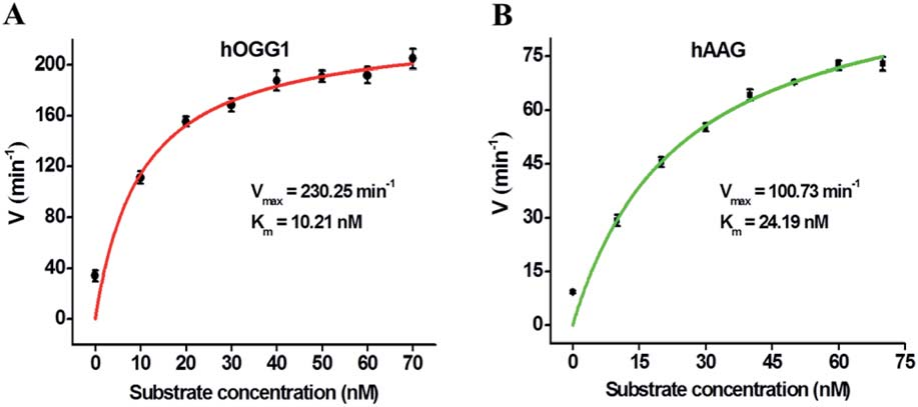
(A) Variance of initial velocity for hOGG1 in response to different concentrations of DNA substrate (*i.e.*, dumbbell probe). The 0.1 U μL^−1^ hOGG1 and 0.5 U μL^−1^ APE1 were used in the experiments. (B) Variance of initial velocity for hAAG in response to different concentrations of DNA substrate (*i.e.*, dumbbell probe). The 0.1 U μL^−1^ hAAG and 0.5 U μL^−1^ APE1 were used in the experiments. Error bars represent the standard deviations of three independent experiments.

We further used the proposed method to measure the enzyme kinetic parameters of hOGG1 and hAAG, respectively. In the presence of 0.1 U μL^−1^ hOGG1, 0.5 U μL^−1^ APE1, and different concentrations of DNA substrate (*i.e.*, dumbbell probe), the initial velocities (*V*) of hOGG1 were quantified after 2 min hOGG1-catalyzed 8-oxoG excision repair reaction at 37 °C (Fig. 6A). In the presence of 0.1 U μL^−1^ hAAG, 0.5 U μL^−1^ APE1, and different concentrations of dumbbell probe, the initial velocities (*V*) of hAAG were detected after 5-min hAAG-catalyzed deoxyinosine excision repair reaction at 37 °C (Fig. 6B). As shown in Fig. 6A, the initial velocity of hOGG1 enhances correspondingly with the increasing concentration of dumbbell probes (*i.e.*, DNA substrates). According to the Michaelis–Menten equation *V* = *V*_max_[S]/(*K*_m_ + [S]), where *V*_max_ is the maximum initial velocity, [S] is the concentration of dumbbell probe, and *K*_m_ is the Michaelis–Menten constant. *V*_max_ is determined to be 230.25 min^−1^ and *K*_m_ is determined to be 10.21 nM for hOGG1. The resulting *K*_m_ value is consistent with those obtained by gel-based radioactive assay (8.9 nM),^52^ single quantum dot-based nanosensor (10.7 nM),^10^ and the Fok I-assisted signal amplification-based fluorescent assay (12.1 nM).^25^ Similarly, the initial velocity of hAAG increases correspondingly with the increasing concentration of dumbbell probes (Fig. 6B). According to the Michaelis–Menten equation, *V*_max_ is calculated to be 100.73 min^−1^, and *K*_m_ is calculated to be 24.19 nM for hAAG. The obtained *K*_m_ value is consistent with that obtained by radioactive assays (13–25 nM),^53^ and the autocatalytic cleavage-mediated fluorescence recovery-based assay (22.1 nM).^11^ These results demonstrate that the proposed method can be applied to evaluate the kinetic parameters of hOGG1 and hAAG with high accuracy.

**Fig. 7:**
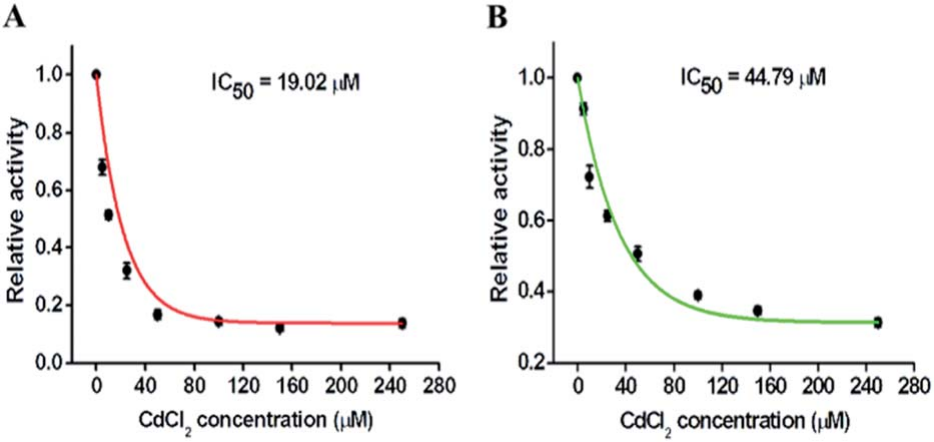
(A) Variance of the relative activity of hOGG1 in response to different concentrations of CdCl_2_. (B) Variance of the relative activity of hAAG in response to different concentrations of CdCl_2_. The 0.1 U μL^−1^ hOGG1, 0.1 U μL^−1^ hAAG, and 0.5 U μL^−1^ APE1 were used in the experiments. Error bars represent the standard deviations of three independent experiments.

Repair glycosylases have been recognized not only as important diagnostic biomarkers, but also as potential therapeutic targets, and screening of their potential inhibitors is critical to anticancer drug discovery and cancer therapy. To demonstrate the feasibility of the proposed method for inhibition assay, we used chromium(ii) chloride (CdCl_2_, a classic inhibitor of repair glycosylases) as a model inhibitor.^54^ CdCl_2_ can inhibit DNA glycosylase activity through two different pathways: (1) Cd^2+^ ion can competitively occupy the same site of hOGG1 that is bound to the DNA substrate, preventing cleavage of the DNA substrate;^40^ (2) Cd^2+^ ion can directly bind the active site (*i.e.*, Zn^2+^-binding site) of hAAG, inactivating the catalytic activity.^55^ As shown in Fig. 7, with an increase in CdCl_2_ concentration from 0 to 250 μM, the relative activities of hOGG1 and hAAG decrease in a concentration-dependent manner. The IC_50_ value (the inhibitory concentration required to reduce the enzyme activity by half) is used to evaluate the inhibition effect of Cd^2+^ on repair glycosylases. According to the fitted calibration curve (Fig. 7A), the IC_50_ value of hOGG1 is calculated to be 19.02 μM, consistent with the values obtained by single quantum dot-based nanosensor (10.93 μM)^10^ and the Fok I-assisted signal amplification-based fluorescent assays (8.86 μM).^25^ Similarly, according to the fitted calibration curve (Fig. 7B), the IC_50_ value of hAAG is calculated to be 44.79 μM, comparable with the value obtained by the radioactive assay (∼100 μM),^55^ and that obtained by the DNA repair-responsive molecular beacon-based fluorescent assay (66.57 μM).^29^ Furthermore, we used human lung adenocarcinoma cell line (A549 cells) as a model to demonstrate the cellular inhibition assay. When the concentration of CdCl_2_ increases from 0 to 300 μM, the relative activities of hOGG1 and hAAG decrease, respectively (Fig. S5†). The IC_50_ values are calculated to be 35.44 μM for hOGG1 and 75.68 μM for hAAG, consistent with the values (19.02 μM for hOGG1 and 44.79 μM for hAAG) obtained by using pure repair glycosylases (Fig. 7). These results demonstrate that the proposed method can be applied for the simultaneous screening of multiple repair glycosylase inhibitors, holding great promise in drug discovery.

**Fig. 8:**
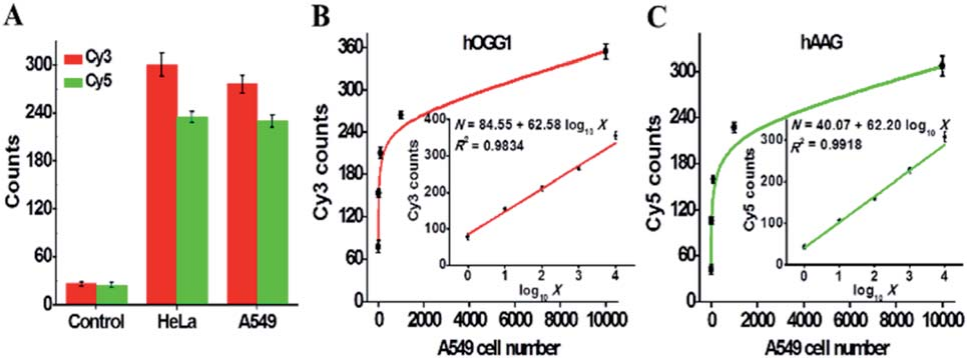
(A) Measurement of Cy3 (red column) and Cy5 (green column) counts in response to the control (extraction buffer only), 1000 HeLa cells, and 1000 A549 cells, respectively. (B) Variance of the Cy3 counts in response to different numbers of A549 cells. The inset shows the linear relationship between the Cy3 counts and the logarithm of A549 cell number from 1 to 10 000 cells. (C) Variance of the Cy5 counts in response to different numbers of A549 cells. The inset shows the linear relationship between the Cy5 counts and the logarithm of A549 cell number from 1 to 10 000 cells. Error bars represent the standard deviations of three independent experiments.

The accurate quantification of repair glycosylases in real samples is of great significance to biomedical research and clinical diagnosis. To demonstrate the capability of the proposed method for potential clinical applications, we used the human cervical carcinoma cell line (HeLa cells) and A549 cells as the models for the simultaneous detection of cellular hOGG1 and hAAG activities (Fig. 8). As shown in Fig. 8A, no distinct Cy3 and Cy5 fluorescence signals are observed in response to the control group with only the extraction buffer. On the other hand, significant Cy3 and Cy5 fluorescence signals are detected in response to HeLa and A549 cells, respectively, indicating that hOGG1 and hAAG are overexpressed in both human cervical and lung cancer cells, which is consistent with previously reported research.^11,15^ Importantly, when the number of A549 cells increases from 1 to 10 000, the Cy3 counts enhance in a dose-dependent manner (Fig. 8B), and a linear correlation is obtained between the Cy3 counts and the logarithm of A549 cell number in the range from 1 to 10 000 cells (inset of Fig. 8B). The regression equation is *N* = 84.55 + 62.58 log_10_ *X* with a correlation coefficient of 0.9834, where *N* is the measured Cy3 count and *X* is the number of A549 cells. The detection limit is directly measured to be 1 cell. The sensitivity of the proposed method is much higher than those of DNA repair-responsive molecular beacon-based fluorescent assay (7 cells)^29^ and single quantum dot-based nanosensor (5 cells),^10^ and it is even comparable to that obtained by the LAMP-based fluorescent assay (1 cell).^15^ These results indicate that the proposed method can be applied to accurately quantify hOGG1 activity in A549 cells. Similarly, when the number of A549 cells increases from 1 to 10 000, the Cy5 counts enhance in a dose-dependent manner (Fig. 8C), and a linear correlation is obtained between the Cy5 counts and the logarithm of the A549 cell number in the range from 1 to 10 000 cells (inset of Fig. 8C). The regression equation is *N* = 40.07 + 62.20 log_10_ *X* with a correlation coefficient of 0.9918, where *N* is the measured Cy5 counts, and *X* is the number of A549 cells. The detection limit is directly measured to be 1 cell. The sensitivity of the proposed method is much higher than that obtained by the DNA repair-responsive molecular beacon-based fluorescent assay (9 cells),^29^ and is comparable to that obtained by the autocatalytic cleavage-mediated fluorescence recovery-based assay (1 cell).^11^ Moreover, we evaluated the recoveries of repair glycosylases by spiking different concentrations of hOGG1 (5 × 10^−7^ − 0.4 U μL^−1^) and hAAG (5 × 10^−7^ − 0.1 U μL^−1^) into normal human serum. As shown in Tables S1 and S2,† the recoveries are determined to be 97.21–107.13% with a relative standard deviation (RSD) of 1.01–2.24% for hOGG1 and 94.97–108.45% with an RSD of 1.36–2.78% for hAAG, consistent with the values (recovery of 99.6–101.0% with an RSD of 0.98–2.34% for hAAG) obtained by the autocatalytic cleavage-mediated fluorescence recovery-based assay.^11^ To verify the results of cellular hOGG1 and hAAG, we used western blotting to analyze the expression levels of repair glycosylases in different parts of HeLa and A549 cells (Fig. S6†). These results demonstrate that the proposed method can be used to accurately quantify multiple repair glycosylases in complex real samples, even at the single-cell level, providing great potential in clinical diagnosis.

## Conclusions

DNA alkylation and oxidation, two of the most important types of genomic damage, are seriously destructive to the maintenance of genome integrity, and studies on repair glycosylases can provide keen insight into the chemistry of DNA lesion repair and the mechanisms of tumorigenesis. In this research, by integrating T7-based transcription with single-molecule detection, we demonstrate for the first time the development of a controlled T7 transcription-driven symmetric amplification cascade machinery for single-molecule detection of multiple repair glycosylases (*i.e.*, hOGG1 and hAAG). In comparison with conventional DNA amplification techniques (*e.g.*, PCR,^30,31^ SDA,^56^ RCA,^24^ EXPAR^33,34^ and EASA^25^), this strategy utilizes *in vitro* T7-based transcription amplification and successive DSN-catalyzed recycling digestion of RNA/DNA hybrids to achieve excellent amplification specificity and high amplification efficiency, effectively eliminating the nonspecific amplifications independent of templates/primers^24,30,31,33,34,56^ and substrates,^25^ and improving the sensitivity and reproducibility in complex environments. Taking advantage of the high accuracy and selectivity of repair glycosylase-catalyzed damaged base excision repair, the high specificity and efficiency of T7 transcription-dependent cycling cascade amplification, and the high signal-to-noise ratio of single-molecule detection, this method exhibits good specificity and high sensitivity with a detection limit of 3.52 × 10^−8^ U μL^−1^ for hOGG1 and 3.55 × 10^−7^ U μL^−1^ for hAAG over a large dynamic range of five orders of magnitude, and it can even quantify cellular repair glycosylases at the single-cell level, superior to most reported repair glycosylase assays.^10,11,22,25,29,50,51^ Moreover, this method can be applied to discriminate different interference enzymes, measure enzymatic kinetic parameters, screen potential inhibitors, determine repair glycosylase activities in human serum, and even be extended to monitor other repair glycosylases by simply changing the specific damaged bases in the dumbbell probe. Given these favorable attributes of operational simplicity, excellent specificity, high sensitivity, practical generality, and potential integration with existing technologies, we believe that this method will provide a new paradigm for *in vitro* transcription engineering, offering a facile and robust platform for the detection of multiple repair glycosylases, with broad applications in genomic repair-related biomedical research, drug discovery, and clinical diagnosis.

## Experimental section

### Chemicals and materials

Human 8-oxoguanine-DNA glycosylase (hOGG1), 10× NEBuffer 2 (500 mM sodium chloride (NaCl), 100 mM trizma hydrochloride (Tris–HCl), 100 mM magnesium chloride (MgCl_2_), 10 mM DL-dithiothreitol (DTT), pH 7.9), human alkyladenine DNA glycosylase (hAAG), 10× ThermoPol reaction buffer pack (200 mM Tris–HCl, 100 mM ammonium sulfate ((NH_4_)_2_SO_4_), 100 mM potassium chloride (KCl), 20 mM magnesium sulfate (MgSO_4_), 1% Triton X-100, pH 8.8), UDG, human apurinic/apyrimidinic endonuclease (APE1), 10× NEBuffer 4 (500 mM potassium acetate (KAc), 200 mM tris-acetate (Tris-Ac), 100 mM magnesium acetate (Mg(Ac)_2_), 10 mM DTT, pH 7.9), T7 RNA polymerase, 10× RNAPol reaction buffer (400 mM Tris–HCl, 60 mM MgCl_2_, 20 mM spermidine, 100 mM DTT, pH 7.9), and ribonucleotide solution set (*i.e.*, ATP, UTP, GTP and CTP) were purchased from New England Biolabs (Ipswich, MA, USA). DSN and 10× DSN master buffer (500 mM Tris–HCl, 50 mM MgCl_2_ and 10 mM DTT, pH 8.0) were obtained from Evrogen Joint Stock Company (Moscow, Russia). Diethylpyrocarbonate (DEPC)-treated water (RNase free) and SYBR Gold were obtained from Invitrogen Corporation (Carlsbad, California, USA). Chromium(ii) chloride (CdCl_2_) and BSA were purchased from Sigma-Aldrich Company (St. Louis, MO, USA). RNase inhibitor was bought from Sangon Biological Engineering Technology & Services Co. Ltd. (Shanghai, China). A549 cells were obtained from Cell Bank, Shanghai Institutes for Biological Sciences, Chinese Academy of Sciences (Shanghai, China). All HPLC-purified oligonucleotides (Tables S3 and S4†) were synthesized by Takara Biotechnology Co. Ltd. (Dalian, China).

### Repairing-activated T7 transcription-dependent cycling cascade amplification

All the synthetic oligonucleotides were dissolved in 1× Tris–EDTA buffer (10 mM Tris, 1 mM EDTA, pH 8.0) for the preparation of stock solutions. The dumbbell probes were diluted to 10 μM with the hybridization buffer (1.5 mM MgCl_2_, 10 mM Tris–HCl, pH 8.0), incubated at 95 °C for 5 min, followed by slowly cooling to room temperature over 30 min to fold into perfect hairpin structures. Then, 1 μL of dumbbell probes was added into 20 μL of excision reaction system containing different concentrations of hOGG1 and hAAG, 10 U of APE1, 2 μL of 10× NEBuffer 2, 2 μL of 10× ThermoPol reaction buffer pack, and 2 μL of 10× NEBuffer 4, and incubated at 37 °C for 30 min to carry out the base excision repair. Subsequently, 10 μL of excision products was added into 10 μL of amplification reaction system containing 40 μM NTPs, 30 U of T7 RNA polymerase, 20 U of RNase inhibitor, and 2 μL of 10× RNAPol reaction buffer, and incubated at 37 °C for 40 min to perform the transcription amplification. After the transcription reaction, 0.7 U of DSN, 500 nM of signal probe 1, 500 nM of signal probe 2, and 2 μL of 10× DSN master buffer were added into the above amplification reaction system, and incubated at 55 °C for 40 min to perform the DSN-directed cyclic cleavage of signal probes.

### Electrophoresis analysis and steady-state fluorescence measurements

To analyze the products of excision and transcription amplification reactions, 14% denaturating polyacrylamide gel electrophoresis (PAGE) was carried out in 1× TBE buffer (9 mM Tris–HCl, 9 mM boric acid, 0.2 mM EDTA, pH 7.9) at a 110 V constant voltage for 50 min at room temperature. After gel electrophoresis, SYBR Gold was used as the fluorescent indicator to stain the gels. The stained gels were then visualized by a ChemiDoc MP Imaging system (Hercules, California, USA). For fluorescence measurement, 20 μL of amplification products was diluted to a final volume of 60 μL with ultrapure water. The fluorophores were analyzed by using an illumination source of Epi-green (520–545 nm excitation) and a 577–613 nm filter for Cy3 fluorophore, and an illumination source of Epi-red (625–650 nm excitation) and a 675–725 nm filter for Cy5 fluorophore, respectively. The fluorescence spectra of Cy3 and Cy5 fluorescent molecules were measured by a Hitachi F-7000 fluorescence spectrophotometer (Tokyo, Japan) at an excitation wavelength of 520 and 632 nm, respectively. The fluorescence intensities of Cy3 and Cy5 were recorded at the emission wavelength of 564 and 663 nm, respectively, for data analysis.

### Single-molecule detection and data analysis

For TIRF imaging, the imaging buffer (67 mM glycine-KOH (pH 9.4), 1 mg mL^−1^ Trolox, 50 μg mL^−1^ BSA, and 2.5 mM MgCl_2_) and the oxygen-scavenging buffer (1 mg mL^−1^ glucose oxidase, 0.04% mg mL^−1^ catalase, and 0.4% (w/v) d-glucose) were freshly prepared. Then the reaction products were diluted 400-fold with the above buffers. The 10 μL samples were directly pipetted to the coverslip for fluorescence imaging. The Cy3 and Cy5 fluorophores were simultaneously excited by sapphire 561 nm and 640 nm lasers *via* the total internal reflection, and the photons were collected through an oil-immersion objective (CFI Apochromat TIRF 100×). The fluorescence of Cy3 and Cy5 was separated by a dichroic mirror, and was imaged onto an Andor ixon Ultra 897 EMCCD camera. For data analysis, ImageJ software was used for counting the Cy5 and Cy3 fluorescent molecules.

### Inhibition assay

For the inhibition assay, different concentrations of CdCl_2_ were incubated with 0.1 U μL^−1^ hOGG1 and 0.1 U μL^−1^ hAAG at 37 °C for 20 min, followed by the addition of the excision and amplification reaction systems for the hOGG1 and hAAG assays using the same procedures as described above. The relative activities (RAs) of hOGG1 and hAAG were determined according to the following equation: RA (%) = (*N*_i_ − *N*_0_)/(*N*_t_ − *N*_0_), where *N*_0_ is the Cy3/Cy5 count in the absence of hOGG1/hAAG, *N*_t_ is the Cy3/Cy5 count in the presence of hOGG1 (0.1 U μL^−1^)/hAAG (0.1 U μL^−1^), and *N*_i_ is the Cy3/Cy5 count in the presence of hOGG1/hAAG and CdCl_2_, respectively. The RA was plotted against the concentrations of CdCl_2_, and the IC_50_ value of CdCl_2_ was calculated from the fitting curve.

### Cell culture and preparation of cellular extracts

Human lung adenocarcinoma cell line (A549 cell) and cervical carcinoma cell line (HeLa cell) were cultured in DMEM supplemented with 10% fetal bovine serum and 1% penicillin–streptomycin in a humidified chamber containing 5% CO_2_ at 37 °C. At the exponential phase of growth, the cells were collected with trypsinization, washed twice with ice-cold phosphate buffered saline, and pelleted at 1000 rpm at 4 °C for 5 min. The nuclear extracts were prepared by using a nuclear extract kit (Active Motif, Carlsbad, CA, USA). The obtained supernatants were immediately subjected to hOGG1/hAAG activity assays.

### Western blotting analysis

For western blotting analysis, the rabbit anti-hOGG1 and hAAG polyclonal antibodies (ZIKER-3687R and 2412R, ZIKER Bio, Shenzhen, China) were used against hOGG1 and hAAG expressed in A549 and HeLa cells, respectively. After cancer cells (5 × 10^6^) were collected, hOGG1 and hAAG enzymes were extracted from the nucleus and cytoplasm in A549 and HeLa cells, respectively, using the nuclear extract kit, and the resultant supernatants were analyzed by western blotting. With histone H3 (RLM3038, RuiYing Bio, Wuhan, China) and actin (GB12001, Servicebio, Wuhan, China) as the internal reference proteins, the levels of hOGG1 and hAAG enzymes from different parts of A549 and HeLa cells were evaluated using the western blot detection kit (E-IR-R304A, Elabscience, Wuhan, China). The immune complexes were detected by an excellent chemiluminescent substrate detection kit (E-BC-R347) (Elabscience, Wuhan, China), and the intensities of protein strips were measured by an Epson V300 scanner (Epson, Suwa, Japan) and quantified by Alpha Ease FC software (Alpha Innotech, San. Leandro, CA, USA).

## Author contributions

Li-juan Wang: conceptualization, methodology, investigation, writing – original draft preparation; Le Liang: methodology, investigation; Bing-jie Liu: methodology, investigation; BingHua Jiang: conceptualization, writing – review and editing; Chun-yang Zhang: conceptualization, funding acquisition, writing – review and editing.

## Conflicts of interest

There are no conflicts to declare.

## Supplementary Material

SC-012-D1SC00189B-s001
