# Supplementary material for: A controlled T7 transcription-driven symmetric amplification cascade machinery for single-molecule detection of multiple repair glycosylases
Source: Chem Sci. 2021 Mar 12;12(15):5544–54. doi: 10.1039/d1sc00189b (PMC8179622; doi:10.1039/d1sc00189b)
Supplement: SC-012-D1SC00189B-s001 [file SC-012-D1SC00189B-s001.pdf]

Electronic Supplementary Material (ESI) for Chemical Science.
This journal is © The Royal Society of Chemistry 2021

Electronic Supplementary Information

A controlled T7 transcription-driven symmetric amplification cascade machinery for single-molecule detection of multiple repair glycosylases

Li-juan Wang,[†,a,b] Le Liang,[†,a] Bing-jie Liu,[c] BingHua Jiang*[,c] and Chun-yang Zhang*[,a]

[a] College of Chemistry, Chemical Engineering and Materials Science, Collaborative Innovation Center of Functionalized Probes for Chemical Imaging in Universities of Shandong, Key Laboratory of Molecular and Nano Probes, Ministry of Education, Shandong Provincial Key Laboratory of Clean Production of Fine Chemicals, Shandong Normal University, Jinan 250014, China.

[b] School of Chemistry and Chemical Engineering, Southeast University, Nanjing 211189, China.

[c] Academy of Medical Sciences, Zhengzhou University, Zhengzhou 450000, China.

* Corresponding author. Tel.: +86 0531-86186033; Fax: +86 0531-82615258. E-mail: cyzhang@sdnu.edu.cn, bhjiang@zzu.edu.cn.

[†] These authors contributed equally.

1. Optimization of the amount of T7 RNA polymerase.

In this assay, in the presence of T7 RNA polymerase, T7 promoters can activate the transcription amplification reactions with the unfolded loops 1 and 2 of dumbbell probe as the templates, respectively, inducing efficient transcription of the templates to produce large amounts of reporter probes 1 and 2. Thus, T7 RNA polymerase is crucial for the initiation of T7 transcription-dependent cycling cascade amplification, and the amount of T7 RNA polymerase should be optimized. As shown in Fig. S1, in the presence of hOOG1, the Cy3 fluorescence intensity enhances with the increasing amount of T7 RNA polymerase from 5 to 30 U, and reaches the plateau at the amount of 30 U (Fig. S1, red column). Similarly, in the presence of hAAG, the Cy5 fluorescence intensity improves with the increasing amount of T7 RNA polymerase from 5 to 30 U, and reaches the plateau at the amount of 30 U (Fig. S1, green column). Therefore, 30 U is selected as the optimal amount of T7 RNA polymerase.

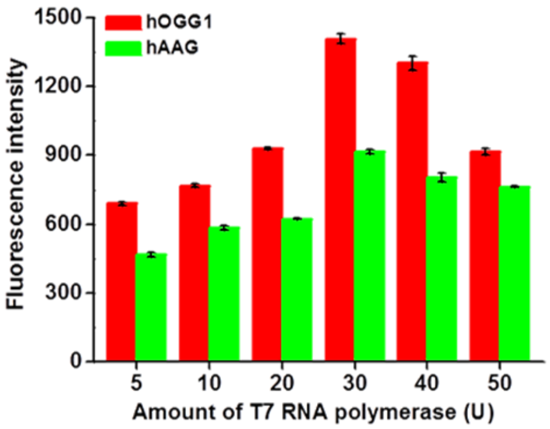

Fig. S1 Variance of Cy3 (red column) and Cy5 (green column) fluorescence intensities with different amounts of T7 RNA polymerase in the range from 5 to 50 U, respectively. Error bars represent the standard deviations of three independent experiments.

2. Optimization of the amount of nuclease DSN.

In this assay, nuclease DSN is responsible for the recycling digestion of signal probes in RNA/DNA heteroduplexes to liberate large numbers of Cy3 and Cy5 fluorophores. Therefore, the amount of DSN can directly affect the digestion efficiency of signal probes and the detection sensitivity of the proposed method. As shown in Fig. S2, we optimized the amount of DSN. In the presence of hOOG1, the Cy3 fluorescence intensity enhances gradually with the increasing amount of DSN from 0.1 to 0.7 U, and reaches the plateau beyond the amount of 0.7 U. Similarly, in the presence of hAAG, the Cy5 fluorescence intensity improves with the increasing amount of DSN from 0.1 to 0.7 U, followed by decrease beyond the amount of 0.7 U. Therefore, 0.7 U is chosen as the optimal amount of DSN in the subsequent research.

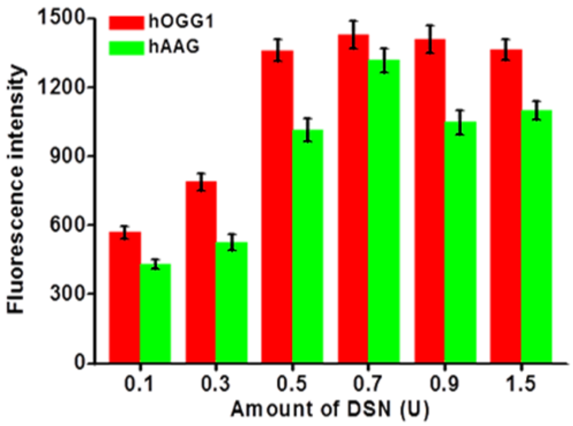

Fig. S2 Variance of Cy3 (red column) and Cy5 (green column) fluorescence intensities with different amounts of DSN in the range from 0.1 to 1.5 U. Error bars represent the standard deviations of three independent experiments.

3. Optimization of different types of signal probes.

To achieve the low background fluorescence of signal probes and the high digestion efficiency of DSN toward signal probes, we synthesized different types of reporter probes and signal probes (Table S4), and performed the optimization experiments (Fig. S3). As shown in Figs. S3A and 3B,

the fluorescence resonance energy transfer (FRET) efficiency between fluorescein and quencher increases in the order of SP1 > SP1-3 > SP1-2 and SP2 > SP2-3 > SP2-2, respectively, consistent with the reported research that the distance between fluorescein and quencher should be as short as possible for the achievement of efficient FRET and the FRET pair (e.g., Cy3 / BHQ2 and Cy5 / BHQ3) should be modified at both ends of sequences.[1-4] With the addition of DSN and reporter probes, the optimization of signal probes were carried out. As shown in Figs. S3C and 3D, comparable Cy3 and Cy5 fluorescence intensities are detected in the presence of different signal probes (SP1 or SP1-3) + reporter probe 1 and different signal probes (SP2 or SP2-3) + reporter probe 2, respectively, indicating that the nuclease DSN exhibits high digestion efficiency toward the DNA-RNA hybrids with 10-bp sequences, not limited to the DNA-RNA hybrids with >15-bp sequences as described in previous researches.[5, 6] Therefore, the signal probes 1 and 2 are used in the subsequent research.

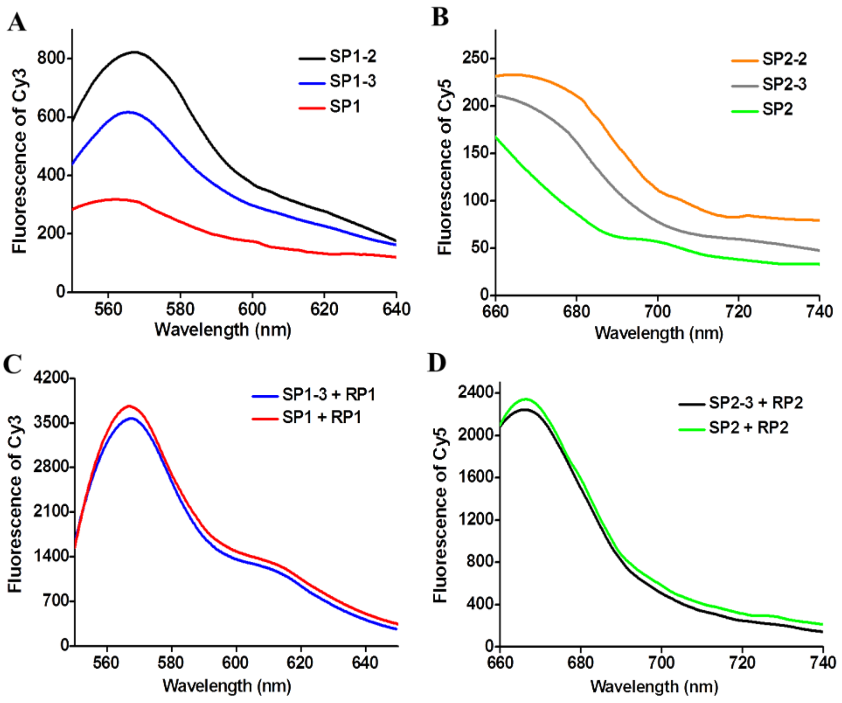

Fig. S3 (A) Variance of Cy3 fluorescence intensities with different signal probes (SP1, SP1-2, or

SP1-3). (B) Variance of Cy5 fluorescence intensities with different signal probes (SP2, SP2-2, or SP2-3). (C) Variance of Cy3 fluorescence intensities in response to different signal probes (SP1 or SP1-3) + reporter probe 1. (D) Variance of Cy5 fluorescence intensities in response to different signal probes (SP2 or SP2-3) + reporter probe 2. In Figs. 3A and 3B, 500 nM signal probes were used in the experiments. In Figs. 3C and 3D, 500 nM signal probes, 1500 nM reporter probes, and 0.7 U of DSN were used in the experiments. Error bars represent the standard deviations of three independent experiments.

4. Optimization of concentration of signal probes.

In this assay, the signal probes 1 and 2 can hybridize with the reporter probes 1 and 2 generated from the T7-based transcription amplification reactions to form two RNA/DNA heteroduplexes 1 and 2, respectively, inducing DSN-directed recycling digestion of signal probes 1 and 2 in heteroduplexes to liberate large numbers of Cy3 and Cy5 fluorophores. Thus, the concentrations of signal probes are directly associated with the detection sensitivity of the proposed method and should be optimized. As shown in Fig. S4, in the presence of hOOG1, the Cy3 fluorescence intensity enhances with the increasing concentration of signal probes, and reaches the maximum value at 500 nM. While in the presence of hAAG, the Cy5 fluorescence intensity improves with the increasing concentration of signal probe from 100 to 500 nM, followed by decrease beyond the concentration of 500 nM. Thus, 500 nM signal probes 1 and 500 nM signal probes 2 are used in the subsequent research.

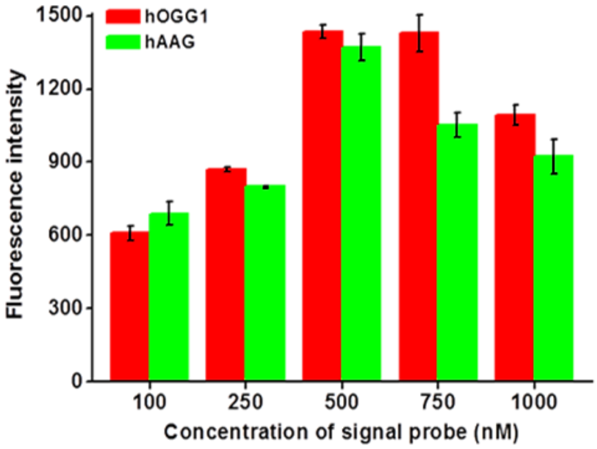

Fig. S4 Variances of Cy3 (red column) and Cy5 (green column) fluorescence intensities with different concentrations of signal probes in the range from 100 to 1000 nM, respectively. Error bars represent the standard deviations of three independent experiments.

5. Inhibition analysis of multiple repair glycosylases assay in cells.

To investigate the capability of the proposed method for cellular inhibition assay, we used human lung adenocarcinoma cell line (A549 cells) as a model. For inhibition assay, 10000 A549 cells were incubated with different concentrations of $CdCl_2$ in phosphate-buffered saline (PBS) at 37 °C for 30 min.[7] Then the cells were collected with trypsinization, washed twice with PBS, and pelleted at 1000 rpm at 4 °C for 5 min. The cellular extracts were prepared with a nuclear extract kit (Active Motif, Carlsbad, CA, U.S.A.), and the obtained supernatants were immediately subjected to hOGG1 / hAAG assays. As shown in Fig. S5, when the concentration of $CdCl_2$ increases from 0 to 300 μM, the relative activities of hOGG1 and hAAG decrease in a concentration-dependent manner, respectively. The IC_{50} values are calculated to be 35.44 μM for hOGG1 and 75.68 μM for hAAG, consistent with the values (19.02 μM for hOGG1 and 44.79 μM for hAAG) obtained by using pure repair glycosylases (Fig. 7). These results demonstrate that this

method can be used to simultaneously screen multiple repair glycosylases inhibitors, holding great potentials in drug discovery.

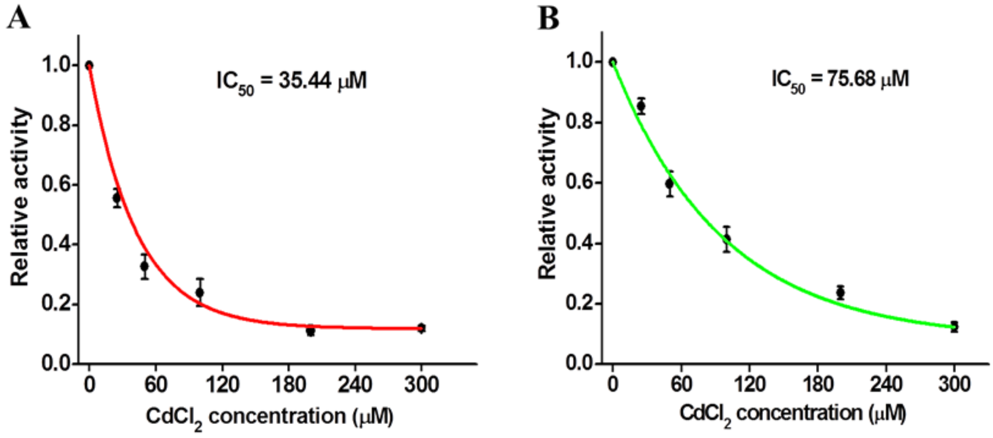

Fig. S5 (A) Variance of the relative activity of hOGG1 from 10000 A549 cells in response to different concentrations of $CdCl_2$. (B) Variance of the relative activity of hAAG from 10000 A549 cells in response to different concentrations of $CdCl_2$. The 0.5 U μL^{-1} APE1 was used in the experiments. Error bars represent the standard deviations of three independent experiments.

6. Cellular repair glycosylases analysis with western blotting.

To verify the detection results of cellular repair glycosylases (i.e., hOGG1 and hAAG), we used western blotting to analyze the protein extracts in cytoplasm and nucleus from different cancer cells (i.e., A549 and HeLa cells) (Fig. S6). As shown in Figs. S6A and S6C, we investigated the expression levels of hOGG1 and hAAG enzymes from A549 and HeLa cells, respectively, using the rabbit anti-hOGG1 and anti-hAAG polyclonal antibodies. Distinct bands (~39 KDa for hOGG1 and ~33 KDa for hAAG) are observed in the presence of nucleus (Fig. S6A, lanes 1 and 2) from A549 and HeLa cells, respectively, while only weak bands are observed in the presence of cytoplasm extracts (Fig. S6C, lanes 1 and 2), suggesting that the expressions of hOGG1 and hAAG in nucleus are much higher than that in cytoplasm. Moreover, the intensities of above

bands are semi-quantified by densitometry. As shown in Figs. S6B and S6D, with the internal reference proteins (i.e., histone H3 and actin) as the controls, the values of relative intensities in response to nucleus and cytoplasm extracts from A549 and HeLa cells are obtained, respectively. In A549 cells, the levels of hOGG1 and hAAG proteins in nucleus extracts are 6.0- and 7.8-fold higher than that in cytoplasm extracts. In HeLa cells, the levels of hOGG1 and hAAG proteins in nucleus extracts are 3.2- and 5.9-fold higher than that in cytoplasm extracts. These results suggest that hOGG1 and hAAG enzymes are highly expressed in the nuclei of cancer cells (e.g., A549 and HeLa cells), consistent with the previous reports.[8-10]

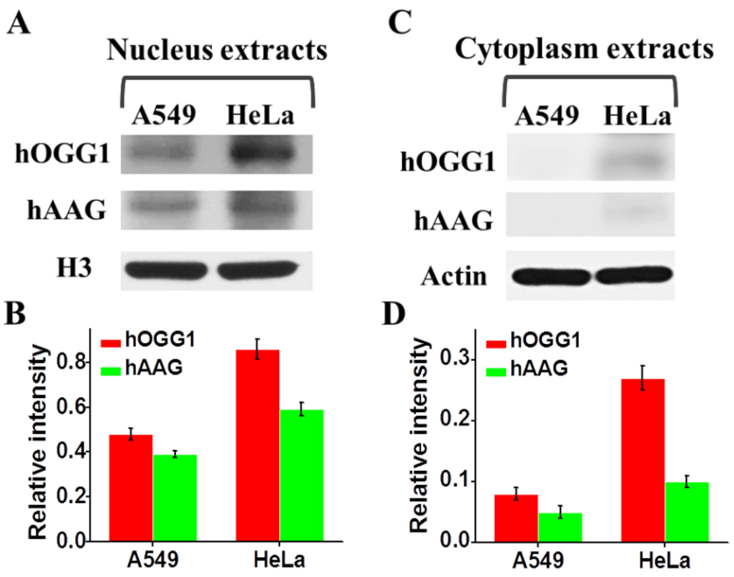

Fig. S6 (A) Analysis of hOGG1 and hAAG expressions in nucleus extracts from A549 and HeLa cells, respectively. (B) Relative intensities of bands in Fig. S6A. (C) Analysis of hOGG1 and hAAG expressions in cytoplasm extracts from A549 and HeLa cells, respectively. (D) Relative intensities of bands in Fig. S6B. The relative intensity is the ratio value of I_t / I_i (I_t is the band intensity in response to target sample (i.e., nucleus and cytoplasm extracts), and I_i is the band intensity in response to the internal reference protein (i.e., H3 and actin)). Error bars represent the standard deviations of three independent experiments.

7. Simultaneous detection of multiple repair glycosylases in the spiked human serum.

To further verify the feasibility of the proposed method for real sample analysis, we measured the recoveries of multiple repair glycosylases by spiking different concentrations of hOGG1 (5×10^{-7} - 0.4 U μL^{-1}) and hAAG (5×10^{-7} - 0.1 U μL^{-1}) into 10% human serum. As shown in Table S1, the recovery is calculated to be 97.21 - 107.13% with a relative standard deviation (RSD) of 1.01 - 2.24% for hOGG1. Similarly, as shown in Table S2, the recovery is calculated to be 94.97 - 108.45% with a RSD of 1.36 - 2.78% for hAAG, consistent with the value (recovery of 99.6 - 101.0% with a RSD of 0.98 - 2.34%) for hAAG obtained by the autocatalytic cleavage-mediated fluorescence recovery-based assay[2] and the value (recovery of 96.6 - 106.0% and RSD of 1.53 - 5.38%) for hAAG obtained by base-excision repair-mediated triple amplification-based fluorescent assay.[11] These results demonstrate that the proposed method can be applied for simultaneous detection of multiple repair glycosylases in complex real samples.

Table S1. Recovery studies by spiking hOGG1 into 10% human serum.

Sample	Added (U μL^{-1})	Measured (U μL^{-1})	Recovery (%)	RSD (%)
1	5×10^{-7}	5.31×10^{-7}	106.22	2.12
2	1×10^{-6}	1.07×10^{-6}	107.13	2.24
3	1×10^{-4}	1.01×10^{-4}	101.15	1.69
4	1×10^{-2}	0.97×10^{-2}	97.21	1.13
5	0.4	0.39	97.53	1.01

Table S2. Recovery studies by spiking hAAG into 10% human serum.

Sample	Added (U μL^{-1})	Measured (U μL^{-1})	Recovery (%)	RSD (%)
1	5.0×10^{-7}	5.42×10^{-7}	108.45	2.78
2	1.0×10^{-6}	1.05×10^{-6}	105.07	1.82
3	1.0×10^{-4}	1.08×10^{-4}	108.19	2.24
4	1.0×10^{-2}	0.95×10^{-2}	95.38	1.36
5	0.1	0.09	94.97	1.85

8. The sequences of the synthesized oligonucleotides.

Table S3. Sequences of the oligonucleotides [α]

note	sequences (5'-3')
dumbbell probe	G*TA ATA CGA CTC ACT ATA GGG* ITA ATA CTA TCT CTT ATC CCT ATA GTG AGT CGT ATT ACC *TAA TAC GAC TCA CTA TAG GG*O GTG TAT CTC TTT CAC CCC CTA TAG TGA GTC GTA TTA G
signal probe 1	Cy3-TAT CTC TTT C-BHQ2
signal probe 2	Cy5-TAC TAT CTC T-BHQ3

[α] In dumbbell probe, the italicized regions indicate the T7 promoter sequence, and the underlined letters "I" and "O" indicate the damaged bases deoxyinosine and 8-oxoG, respectively. In signal probe 1, the underlined bases "T" and "C" indicate the modifications of a fluorescein (Cy3) at 5′ end and a black hole quencher 2 (BHQ2) at 3′ end, respectively. In signal probe 2, the underlined base "T" indicates the modifications of a fluorescein (Cy5) at 5′ end and a black hole quencher 3

(BHQ3) at 3′ end, respectively.

Table S4. Sequences of the oligonucleotides [α]

note	sequences (5'-3')
signal probe 1-2	GTG TAT(Cy3) CTC TTT CAC CCC C-BHQ2
signal probe 2-2	TAA TAC T(Cy5)AT CTC TTA TCC C-BHQ3
signal probe 1-3	Cy3-GTG TAT CTC TTT CAC CCC C-BHQ2
signal probe 2-3	Cy5-TAA TAC TAT CTC TTA TCC C-BHQ3
reporter probe 1	GGG GGU GAA AGA GAU ACA C
reporter probe 2	GGG AUA AGA GAU AGU AUU A

[α] In signal probe 1-2, the underlined bases “T” and “C” indicate the modifications of a fluorescein (Cy3) and a black hole quencher 2 (BHQ2), respectively. In signal probes 2-2 and 2-3, the underlined bases “T” and “C” indicate the modifications of a Cy5 and a BHQ3, respectively. In signal probe 1-3, the underlined bases “G” and “C” indicate the modifications of a Cy3 at 5′ end and a BHQ2 at 3′ end, respectively.

References

1. L.-j. Wang, M. Ren, Q. Zhang, B. Tang and C.-y. Zhang, *Anal. Chem.*, 2017, **89**, 4488-4494.
2. L.-j. Wang, M.-l. Luo, X.-y. Yang, X.-f. Li, Y. Wu and C.-y. Zhang, *Theranostics*, 2019, **9**, 4450-4460.
3. G. Zhu, K. Yang and C.-y. Zhang, *Biosens. Bioelectron.*, 2013, **49**, 170-175.
4. G. Zhu, Y. Li and C.-y. Zhang, *Chem. Commun.*, 2014, **50**, 572-574.

5. D. A. Shagin, D. V. Rebrikov, V. B. Kozhemyako, I. M. Altshuler, A. S. Shcheglov, P. A. Zhulidov, E. A. Bogdanova, D. B. Staroverov, V. A. Rasskazov and S. Lukyanov, *Genome Res.*, 2002, **12**, 1935-1942.
6. F. Ma, W.-j. Liu, L. Liang, B. Tang and C.-Y. Zhang, *Chem. Commun.*, 2018, **54**, 2413-2416.
7. A. Bravard, M. Vacher, B. Gouget, A. Coutant, F. H. de Boisferon, S. Marsin, S. Chevillard and J. P. Radicella, *Mol. Cell. Biol.*, 2006, **26**, 7430-7436.
8. J. Hu, M.-h. Liu, Y. Li, B. Tang and C.-y. Zhang, *Chem. Sci.*, 2018, **9**, 712-720.
9. Y. Zhang, C.-c. Li, B. Tang and C.-y. Zhang, *Anal. Chem.*, 2017, **89**, 7684-7692.
10. L.-j. Wang, Y.-y. Lu and C.-y. Zhang, *Chem. Sci.*, 2020, **11**, 587-595.
11. H. Zhang, L. Wang, Y. Xie, X. Zuo, H. Chen and X. Chen, *Analyst*, 2019, **144**, 3064-3071.
